# Intranasal administration of inactivated avian influenza virus of H5N1 subtype vaccine-induced systemic immune response in chicken and mice

**DOI:** 10.14202/vetworld.2018.221-226

**Published:** 2018-02-20

**Authors:** I N. Suartha, G. A. A. Suartini, I W. Wirata, N. M. A. R. K. Dewi, G. N. N. Putra, G. A. Y. Kencana, G. N. Mahardika

**Affiliations:** 1 Department of Internal Medicine, Animal Hospital, Faculty of Veterinary Medicine Udayana University, Jl. Sesetan-Markisa 6, Denpasar 80226, Bali, Indonesia; 2 Department of Biochemistry, Faculty of Veterinary Medicine Udayana University, Jl. Sudirman, Denpasar 80225, Bali, Indonesia; 3 Department of Animal Biomedical and Molecular Biology Laboratory Faculty of Veterinary Medicine Udayana University, Jl. Sesetan-Markisa 6, Denpasar 80226, Bali, Indonesia; 4 Department of Virology, Faculty of Veterinary Medicine Udayana University, Jl. Sudirman, Denpasar 80225, Bali, Indonesia

**Keywords:** inactivated vaccine, influenza-H5N1, ISCOMS, Inmunair, intranasal

## Abstract

**Aim:**

The need for non-parenteral administration of inactivated avian influenza virus of H5N1 subtype (AIV-H5N1) vaccine is paramount. Here, we provide preliminary data on the immune response of chicken and mice after intranasal administration of AIV-H5N1-inactivated vaccine with ISCOMS, Inmunair (INM), and combined ISCOMS and INM as an adjuvant.

**Materials and Methods:**

The AIV isolate of A/Chicken/Denpasar/01/2004 (H5N1) was cultivated in specific pathogen-free chicken eggs and inactivated with formaldehyde. The vaccine preparation was added with those adjuvants for intranasal administration and aluminum hydroxide for subcutaneous injection. The chicken and mouse were vaccinated at the age of 3 weeks or 1 month and repeated 2 weeks thereafter. In one experiment, chicken was injected with Newcastle disease virus (NDV) at the same time with AIV vaccine. The sera were collected at one (serum 1) and 2 w (serum 2) after booster vaccination. The anti-AIV-H5 and NDV antibodies in chicken sera were detected using hemagglutination inhibition (HI) assay. Mouse IgG anti-AIV-H5N1 antibody was detected using ELISA.

**Results:**

The result shows that the geometric mean titers (GMTs) of chicken sera of intranasal vaccinated with inactivated AIV-H5N1 vaccine with mixed ISCOM- INM as adjuvant were <2^0.0^ and 2^2.7^ unit HI-unit (HIU) in serum 1 and serum 2, respectively. The GMTs of the positive control group were 2^3.7^ and 2^5.7^ HIU in serum 1 and serum 2, respectively. The result of the second experiment shows that IgG anti-AIV-H5N1 was detected in mouse sera. In the third experiment, the GMTs of anti-NDV in chicken vaccinated subsequently with inactivated NDV vaccine and AIV-H5N1 with mixed ISCOMS-INM administrated intranasally and aluminum hydroxide adjuvant administrated through subcutaneous injection as well as positive control group receiving NDV vaccine only were 2^8.0^, 2^8.0^, and 2^7.4^ HIU in serum 1 while were 2^9.6^, 2^9.2^, and 2^8.2^ HIU in serum 2, respectively.

**Conclusion:**

Intranasal administration of inactivated AIV-H5N1 vaccine-induced a systemic immune response in chicken and mice after adding ISCOMS and/or INM as adjuvants. The adjuvant and the intranasal administration caused no immunosuppressive effect on the chicken immune response to NDV vaccine.

## Introduction

Highly pathogenic avian influenza viruses of the subtype H5N1 (HPAIV-H5N1) have circulated continuously in Asia, Europe, and Africa since 2003 [1]. The viruses constantly undergo genetic drift and shift that permanently threats poultry industry and human health [2]. HPAIV-H5N1 is also responsible for human fatalities, with Indonesia having the highest fatality rate in the world, until a recent increase in the number of human cases in Egypt [3]. Fortunately, many strains have so far shown only limited or unsustainable human-to-human transmission [4]. Human infection is believed as a result of transmission from infected poultry [5].

To reduce the economic loss due to its infection, vaccination is widely implemented, especially in Indonesia, China, Vietnam, and Egypt [6]. Over 95% of HPAIV vaccine applied all over the world is oil-emulsified, inactivated whole AIV vaccines [6]. A disadvantage of the inactive vaccine is that it must be administered parenterally to induce systemic immune response [7]. Some disadvantages of the inactivated vaccine are that it causes stress and induces minimum surface immune response [7]. The surface immune response is beneficial to prevent the entry of infectious agents. We believe that the lack of immunity in the mucosa allows the HPAIV to replicate and shed in the dropping and stool of vaccinated birds on challenge [6]. This phenomenon is known as the vaccine masking effect [8].

The need for non-parenteral administration of inactivated AIV-H5N1 vaccine is paramount. We tried ISCOMS and INM in this study. ISCOMS is stable complex which contains cholesterol, phospholipid, saponin Quill A from *Quillaja Saponaria* Molina plant [9,10]. This adjuvant effectively presents foreign substance to antigen presenting cells and stimulates the production of cytokine and costimulatory factors [11,12]. ISCOMS has been reported to induce humoral local and systemic immune response as well as cell-mediated immunity (CMI) with low antigen quantity [13,14].

INM contains a mixed suspension of inactive *Propionibacterium acne* with lipopolysaccharide (LPS) and is marketed as an immune booster in animal. Inactivated *P. acne* - the causative agent of acne in human [15] - together with LPS has been indicated as humoral and CMI stimulator [16]. The bacteria could substitute *Mycobacterium tuberculosis* in traditional Freund’s Complete Adjuvant [7] for the use beyond the laboratory. LPS is also a strong passive immunity inducer [17]. Some data have demonstrated that *Propionibacterium* sp. activates the mononuclear phagocytes, stimulates inflammatory mediator secretion, and activates T and B lymphocytes [18,19].

Here, we provide preliminary data on the systemic immune response of chicken and mice following nasal drop administration of inactivated AIV-H5N1 vaccine.

## Materials and Methods

### Ethical approval

Ethical clearance for this experiment was provided by the Committee of the Use of Animal in Experiment of the Faculty of Veterinary Medicine Udayana University.

### Laboratory safety

All laboratories work with the live virus was conducted in an isolated room with inlet and outlet air filtered with HEPA filter and equipped with biosafety cabinet class III (BSC-III) with negative pressure. All waste materials were autoclaved inside the room. All staffs were equipped with personal protective equipment.

### Vaccines and animal experiment

As the vaccine seeds, the isolate of influenza A virus (A/Chicken/Denpasar/01/2004(H5N1)) was used in this experiment. The sequences of hemagglutinin and neuraminidase of the isolate are available in GenBank with the Accession No. DQ644955 and KR987715. The isolates were cultivated and titrated in specific pathogen-free (SPF) chicken eggs. The end concentration was 10^8^ 50% egg infectious dose (EID_50_) per 250 µL suspension. The seed virus was inactivated with 0.01% formaldehyde (Merck) and stirred overnight in BSC III cabinet. The treated vaccine preparation was sampled 5 times and injected into SPF eggs to check residual infectious virus. All vaccine preparation had to be negative for infectious virus. Before the administration of vaccine, the preparation was emulsified with an equal volume of selected adjuvant. For intranasal administration, the adjuvants were ISCOMS-AbISCO-300 (Isconova AB), INM 17.5 (Laboratorios Calier), and combined ISCOMS-INM. For subcutaneous injection, the vaccine was added with aluminum hydroxide (Sigma-Aldrich) as an adjuvant. The antigen content of intranasal vaccine preparation was 0.5×10^8^ EID_50_ per 250 µL while for subcutaneous injection was 10^8^ EID_50_ per 500 µL. Commercial inactivated Newcastle disease virus (NDV) vaccine was the product of PT Medion, Bandung, Indonesia. The vaccine was carefully administrated using intranasal drop of the total volume of 250 µL per animal or injected subcutaneously at the backside of the neck of 500 µL per animal.

In the first experiment, six chickens of 3 weeks old were grouped and vaccinated with various vaccine formulations as mentioned above. Booster vaccination was given 2 weeks thereafter. Sera of three chickens were collected at 1 week (serum 1) and 2 weeks (serum 2) after the booster.

In the second experiment, six mice of 1-month-old were grouped and vaccinated with various vaccine formulations as above. Booster vaccination was given 2 weeks thereafter. Sera of three mice were collected at 2 weeks (serum 1) and 2 weeks (serum 2) after the booster. ELISA IgG was conducted from sera. The sera were diluted 1:10 with phosphate-buffered saline (PBS).

In the third experiment, five chickens of 3 weeks old were grouped and vaccinated with various vaccine regiments as above. Booster vaccination was given 2 weeks thereafter. All chickens were vaccinated with inactivated NDV vaccine simultaneously. Sera of all chickens were collected at 2 weeks (serum 1) and 2 weeks (serum 2) after the booster. Anti-NDV antibody was titrated from sera.

### ELISA antigen

As ELISA antigen, inactivated AIV-H5N1 was purified using following procedure. The inactivated AIV-H5N1 preparation was add with an equal volume of 1% chicken red blood cells and stirred for 1 h. The mixture was then centrifuged at 1000 rpm for 1 min. The supernatant was discarded. The RBC and attached virus were suspended and washed 3 times with cold PBS. Receptor destroying enzyme (Sigma-Aldrich) in pre-warmed PBS was added to the mixture after the third wash and stirred overnight. The supernatant was collected and heated at 56°C for 1 h. ELISA-negative antigen was generated by mixing CBRC with PBS (without virus) and treated as above.

### Hemagglutination inhibition (HI) assay

The HI assay to detect anti-AIV-H5 and NDV antibody was conducted following OIE protocol [20]. Antibody titer is expressed in HI unit (HIU) hereinafter.

### Mouse IgG ELISA

The mouse sera were diluted 10 times with PBS. Fifty microliters of purified inactivated AIV-H5N1 diluted in carbonate buffer pH 9.6 were coated in each well of ELISA plate in the refrigerator overnight. After 3 times wash with PBS-tween, the plate was blocked with BSA-PBS/tween for 1 h. After washing, the diluted mouse serum was added to each well for 1 h. The well then added with anti-mouse IgG conjugated with HRPO (ThermoFisher Scientific). Color development was conducted by adding 2,2’-Azinobis [3-ethylbenzothiazoline-6-sulfonic acid]-diammonium salt in substrate solution (24.3 mL of 0.1M citric acid to 25.7 mL of 0.2M dibasic sodium phosphate in the final volume of 100 mL) with 10 uL H_2_O_2_ 30%. The color development was stopped using 1% SDS. Control positive was produced using mouse IgG (ThermoFisher) as coated antigen in phosphate buffer of pH 6.8. Control negative was created using ELISA-negative antigen as described above. The ELISA plate was read in absorbance at 410 nm and 650 nm. Samples were judged positive if the optical density were >5 times OD of the negative control.

## Result

GMT of anti-AIV-H5 antibody in the serum of chicken following vaccination with inactivated AIV vaccine with various adjuvants through nasal drop and subcutaneous injection is presented in Table-1. The result shows that the GMTs of chicken sera intranasally vaccinated with inactivated AIV-H5N1 vaccine with mixed ISCOM, INM, and ISCOM-INM as adjuvant were 2^1.7^, <2^0.0^, and <2^0.0^ at 1 week (serum 1) and 2^2.0^, 2^2.7^, and 2^2.7^ HIU at 2 weeks (serum 2) after booster vaccination, respectively. The GMTs of the positive control group receiving inactivated AIV-H5N1 vaccine with aluminum hydroxide as adjuvant were 2^3.7^ and 2^5.7^ HIU in serum 1 and serum 2, respectively. The GMTs of the negative control group were <2^0.0^ HIU in both serum collection times.

**Table-1:** GMT (−log 2 HI) of anti-AIV-H5 antibody in the serum of chicken following vaccination with inactivated AIV vaccine with various adjuvants through intranasal administration and subcutaneous injection.

Adjuvant	Administration	Animal number	Serum HI titer		

			Serum 1	Serum 2
ISC	Intranasal	1	2	3
		2	2	3
		3	1	2
		GMT	1.7	2.7
INM	Intranasal	1	0	2
		2	0	2
		3	0	2
		GMT	0	2
ISCINM	Intranasal	1	0	3
		2	0	2
		3	0	3
		GMT	0	2.7
AH	SC	1	5	6
		2	4	6
		3	2	5
		GMT	3.7	5.7
Control		1	0	0
		2	0	0
		3	0	0
		GMT	0	0

ISC=ISCOMS; INM=Inmunair; AH=Aluminum hydroxide. Vaccination was conducted at 3 weeks old and booster vaccination was given 2 weeks thereafter. Serum was collected at 1 (serum 1) and 2 (serum 2) weeks after booster vaccination

IgG of anti-AIV H5N1 antibody detection in serum of mice following nasal drop administration of inactivated AIV-H5N1 various adjuvants is shown in Table-2. The result shows that IgG was detected in all treatment groups including AH-SC positive control, except in INM adjuvant group. All mice in the negative control group were negative in IgA and IgG ELISA.

**Table-2:** IgG detection of anti-AIV H5N1 antibody detection in serum of mice following intranasal administration of inactivated AIV-H5N1 with ISCOMS and Inmunair as an adjuvant.

Adjuvant	Administration	Animal number	IgG ELISA of serum	

			Serum 1	Serum 2
ISC	Intranasal	1	+	+
		2	+	+
		3	−	+
INM	Intranasal	1	−	−
		2	−	−
		3	−	−
ISC-INM	Intranasal	1	+	+
		2	+	+
		3	+	+
AH	SC	1	+	+
		2	+	+
		3	+	+
Control		1	−	−
		2	−	
		3	−	

ISC=ISCOMS, INM=Inmunair, AH=Aluminum hydroxide, +=ELISA positive, −=ELISA negative, ELISA was judged positive if the optical density was more than 5 times higher than negative control; Vaccination was conducted at 1-month-old and booster vaccination was given 2 weeks thereafter. Serum was collected at 1 (serum 1) and 2 (serum 2) weeks after booster vaccination

**Figure-1:**
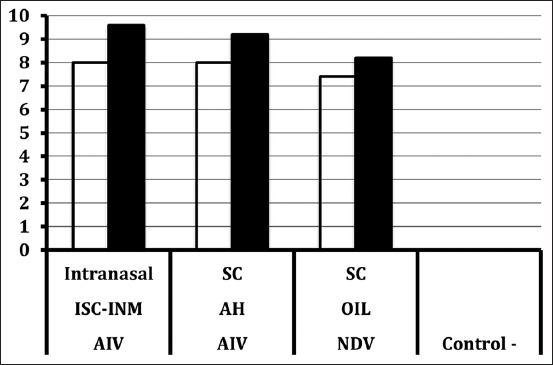
The geometric mean titer of anti-Newcastle disease virus (NDV) antibody titer (−log2 hemagglutination inhibition unit) of chicken sera following administration of commercial inactivated NDV vaccine combined with inactivated AIV-H5N1 AI3G. The NDV and AIV vaccines were given at 3-week-old chicken and booster vaccination was done 2 weeks thereafter. The intranasal vaccine was added with ISCOMS (ISC) and Inmunair. One group of chicken was injected subcutaneously with inactivated AIV-H5N1 vaccine with aluminum hydroxide as an adjuvant. The NDV vaccine was also subcutaneously administered. One group of chicken was left without vaccine for treatment and experiment control.

The GMT of anti-NDV antibody titer of chicken sera following administration of commercial inactivated NDV vaccine combined with inactivated AIV-H5N1 vaccine is graphically presented in Figure-1. The GMTs of anti-NDV vaccinated subsequently with inactivated AIV-H5N1 with mixed ISCOMS-INM administrated intranasally and aluminum hydroxide adjuvant administrated through subcutaneous injection as well as the positive control group receiving NDV vaccine only were 2^8.0^, 2^8.0^, and 2^7.4^ HIU at serum 1 while were 2^9.6^, 2^9.20^, and 2^8.2^ HIU at serum 2, respectively. The GMTs of anti-NDV antibody of the negative control group were <2^0.0^ HIU at both serum collection times.

## Discussion

Vaccination is a choice in reducing the economic impact of HPAIV in poultry industry. An attempt has to be made that the vaccine generates a sterile immune response, in which the vaccinated bird sheds no infectious virus on challenge. Sterile immunity means that the presence of antibodies is sufficient to prevent colonization of mucosal surfaces [21]. The mostly available vaccine against HPAIV-H5N1 is inactivated vaccine which mostly given using intramuscular or subcutaneous injection [6]. This kind of vaccine is proven to produce non-sterile immunity. Such vaccine does generate a protective systemic immune response; however, theoretically, it stimulates minimum mucosal immunity, which is needed to prevent virus shedding on field virus challenge. The threat to human health is unavoidable. Moreover, the ideal vaccine should be administrable in mass population setting, such as through spraying, drinking water, or drop, which should be easier, less time consuming, and avoiding stress for the animal. Contact to mucosa at the first time should stimulate mucosal immune system to produce mucosal antibody of IgA class before recirculation of immune cells to generate systemic immune response [7].

In this experiment, the detection of antibody in serum of chicken and mouse following intranasal administration of vaccine should bring evidence that local administration of vaccine stimulates local immune system before the production of systemic immune response. That the HI titer in serum 1 (1 weeks after booster) is generally lower than serum 2 (2 weeks after booster), it indicates an active antibody production in vaccinated animal. This is a typical figure of immune response of chicken to foreign antigen, which mostly reached maximum immune response in the 3^rd^ to 4^th^ weeks following exposure [7]. The immune responses before booster vaccination were indeed not collected to minimize the stress of animals and to reduce the number of replicates, which meant more animals to be sacrificed. The ethics committee limited our experimental animals due to the number of treatments and the euthanization of the animals in the research plan.

The combination of ISCOMS and INM experimented in this study is novel adjuvant in HPAIV vaccine delivery in chicken. We proof that ISCOMS and INM induce immune response on intranasal administration of inactivated AIV-H5N1 vaccine. There is an evidence found in this study that ISCOMS stimulates immune response both in chicken and in mice while INM failed in mice. The combination of both substances is proposed, especially, to generate feasible adjuvant combination to be used in chicken. The available price of ISCOMS and INM is nowadays not feasible for poultry. New ISCOMS generation should be developed to meet the acceptable price to be used in poultry. That INM alone also stimulates an immune response in chicken, it should be evidence that this adjuvant is worthy to be explored in the near future.

In this experiment, after serum collection, three chicken and mice were euthanized, tracheal and intestinal wash using 1 milliliter PBS were collected, and IgA was detected (not shown). This class of immunoglobulin to HPAIV-H5N1 could be detected only in one chicken and one mouse. The failure to demonstrate convincing IgA response might be due to the improper preparation of tracheal and intestinal wash. Suspension of trachea, lung, and intestine as well as nasal wash might bring better result. Besides that, IgA-expressing lymphocytes specific to AIV-H5N1 might be demonstrated using immunohistochemistry or immune-fluorescence from mucosal scrap or paraffin block. Mucosal IgA has been proven in the lung [22,23] or nasal wash [24-26] of mice following intranasal administration of inactivated or recombinant or modified live vaccine of HPAIV-H5N1.

As biosafety level 3 facility was not available, protection challenge experiment was not conducted. A further experiment is needed to prove that the intranasal administration of HPAIV vaccine provides a good protection as well as reduced virus shedding on the challenge.

## Conclusion

Intranasal administration of inactivated AIV-H5N1 vaccine with uncombined and combined ISCOMS and INM as adjuvants induced a systemic immune response in chicken and mice. The HI-anti H5 antibody of chicken after vaccinated with inactivated vaccine with ISCOMS and/or INM was detectable in 2 weeks after booster, which was lower than traditional SC vaccine. The IgG-anti H5 antibody of mice after vaccinated with inactivated vaccine with ISCOMS and/or INM was detectable in 1 and 2 weeks after the booster. The vaccine formulae caused no immunosuppressive effect on the chicken immune response against NDV vaccine. Further researches are needed to provide evidence that the vaccine formulae provide good and sterile protectivity and to formulate adjuvant, which is feasible to be used in chicken industry.

## Authors’ Contributions

INS, GAAS, and GNM designed this research. IWW, NMARKD, and GNNP prepared the vaccine and conducted an animal experiment. IWW, NMARKD, GNNP, and GAYK collected the samples. INS, GAAS, NMARKD, GNNP, GAYK, and GNM conducted HI and ELISA detection. INS, GAYK, and GNM prepared data set and drafted the manuscript. All authors read and approved the manuscript.
